# Usefulness of oral health assessment performed by multiple professionals using a short video recording acquired with a tablet device

**DOI:** 10.1016/j.jds.2023.11.011

**Published:** 2023-11-28

**Authors:** Yuiko Yanagihara, Hiroyuki Suzuki, Junichi Furuya, Kazuharu Nakagawa, Kanako Yoshimi, Sayaka Seto, Kento Shimizu, Haruka Tohara, Shunsuke Minakuchi

**Affiliations:** a Department of Gerodontology and Oral Rehabilitation, Graduate School of Medical and Dental Sciences, Tokyo Medical and Dental University (TMDU), Tokyo, Japan; b Department of Oral Function Management, Showa University Graduate School of Dentistry, Tokyo, Japan; c Department of Dysphagia Rehabilitation, Graduate School of Medical and Dental Sciences, Tokyo Medical and Dental University (TMDU), Tokyo, Japan; d Department of Nursing, Tokyo Medical and Dental University Hospital, Tokyo, Japan

**Keywords:** Oral health, Nutritional support, Remote consultation, Video recording, Tablet computer, Inpatients

## Abstract

**Background/purpose:**

Appropriate oral health assessment and management can improve the oral health and nutritional status of hospitalized patients. The active participation of dental professionals in the nutritional support team (NST) can help achieve this outcome. However, the participation of dental professionals in NSTs is often limited, indicating the requirement for establishing a remote oral health assessment method. This study aimed to establish a multidisciplinary oral health assessment system using short video recordings with a tablet device.

**Materials and methods:**

Fifty inpatients receiving NST aid at the Tokyo Medical and Dental University Hospital were included in this study. The degree of agreement between the oral health assessment performed at the bedside and using the short video recordings of the oral health acquired with a tablet device by a single dentist and the degree of agreement for evaluations performed using the video recordings between multiple professionals were evaluated. The oral health status was assessed using the Oral Health Assessment Tool (OHAT).

**Results:**

The intraclass correlation coefficient (ICC) of the OHAT total scores for oral health assessment performed at the bedside and using the videos by a single dentist was 0.914 (95% confidence interval [CI], 0.854–0.950). ICCs of the OHAT total scores for the video assessment performed by dentists and multiple professionals were 0.904 (95% CI, 0.838–0.944) and 0.802 (95% CI, 0.676–0.883), respectively.

**Conclusion:**

Comprehensive oral health assessment of patients can be performed by multiple professionals using the short video recordings of the oral health acquired with a tablet device.

## Introduction

Many patients in acute care hospitals require nutritional support, and 13–78% of these patients are malnourished.^1^ Nutrition Support Teams (NST) are multidisciplinary teams comprising physicians, nurses, pharmacists, dietitians, and dentists who provide nutritional support to patients with malnourishment. Several hospitals have established NSTs owing to their effectiveness in improving the nutritional status and quality of life, in addition to shortening hospital stays and reducing medical costs.^2^^,^^3^ Selection of appropriate nutritional intake methods is essential for providing nutritional support as a relationship has been observed between the nutritional intake method and oral health status. For instance, molar occlusal support and tongue function play a role in the selection of oral intake method for patients with dysphagia receiving acute care,^4^ whereas the nutritional intake method is affected by the number of teeth and the requirement for prosthetic treatment in patients with stroke.^5^

More than 50% of patients who require NST aid have poor tongue and oral cleanliness, as well as poor salivary flow. Moreover, approximately 70% of these patients require professional oral care and dental treatment.^6^ Approximately 50% of patients who require NST aid are unable to receive food orally. The number of patients who can receive food orally would increase if an appropriate oral health assessment is performed in addition to a general health assessment.^7^ Nutritional intake improves along with the oral health if oral health management is performed by a multidisciplinary NST with dental professionals.^8^ Thus, multidisciplinary oral care management and appropriate oral health assessment are essential for patients requiring the aid of NST. However, less than 30% of hospitals have a dental department,^9^^,^^10^ and the participation rate of dentists in NSTs is approximately 5%.^11^ Thus, few hospitals have dental professionals who can directly assess the oral health status of patients. In such situations, it is important to establish a system that enables remote assessment of patients’ oral health status by multidisciplinary professionals, such as nurses, who have many opportunities to consult with patients and refer them to dental professionals.

Video recordings can be used to obtain visual information from remote locations. A previous study demonstrated high reliability between the oral health assessment performed by the same evaluator at the bedside and using 10-min video recordings of the participants’ mouths.^12^ However, performing a 10-min video recording in all patients in a clinical setting with various restrictions is challenging. Although shorter videos could be clinically useful for evaluation by multiple professionals, the reliability of this method is unclear. Therefore, this study aimed to examine the feasibility of oral health assessment performed by multiple professionals using short video recordings of the oral health status acquired with a tablet device.

## Materials and methods

### Participants

The inclusion criteria were as follows: patients who were admitted to the Tokyo Medical and Dental University Hospital between January 2022 and January 2023 and underwent oral health assessment performed by a dentist participating in the NST. Patients who had difficulty opening the mouth by the videographer due to impaired consciousness or contractures, and who were unable to take intraoral videos, were excluded. All participants were informed about the use of anonymized medical information and the content of the study and provided the opportunity to refuse participation in the study via the opt-out method. This study was approved by the Ethical Review Committee of the School of Dentistry, Tokyo Medical and Dental University (approval no.: D2021-109).

### Measurements

A dentist performed oral health assessment at the bedside and captured video recordings of the oral health. The recordings were acquired using an iPad Pro (Apple Inc., USA), with sufficient consideration given to personal information. The sequence of the video recording was the lips, frontal view of the anterior teeth, palate and maxillary occlusal surface, mandibular occlusal surface, right and left buccal mucosa, tongue (surface and side), frontal view of the anterior teeth, and lips. Each recording time was approximately 5 s. The oral health of the patients using dentures was recorded without dentures, and the individual dentures were recorded subsequently. The patients were asked about the frequency of denture use and the presence or absence of pain. The duration of the video recording of the oral health status including the dentures, was approximately 1 min. Three examiners, comprising two dentists and one nurse, performed oral health assessment using the video recordings. The dentist who had acquired the recordings was one of the two dentists who performed the oral health assessment using the video recordings. The assessment was conducted at least two weeks after the bedside assessment, as described in a previous study.^12^ The examiners of the assessment were dentists with more than four years of experience as a dentist and certified nurse in dysphagia nursing. Thorough inter-rater calibration was performed prior to commencing the study.

### Outcomes

The total score of the Oral Health Assessment Tool (OHAT),^13^ a comprehensive assessment of the oral health, and scores of the OHAT sub-items were the outcomes assessed in this study. The OHAT is designed to enable non-dental professionals to assess the oral health status and has been adopted by several hospitals. The OHAT sub-items include the lips, tongue, gums and tissues, saliva, natural teeth, dentures, oral cleanliness, and dental pain, each of which is rated on a scale of 0–2. The total score ranged from 0 to 16, with higher scores indicating a poorer oral health.

### Other variables

Basic information, such as the age; sex; body mass index (BMI); history of systemic diseases; level of consciousness; degree of independence; the albumin (Alb), C-reactive protein (CRP), hemoglobin (Hb) levels, and nutritional intake method of the patients, was extracted from the medical records and NST conference information. The Charlson Comorbidity Index (CCI) was used to score systemic diseases with primary diseases and comorbidities.^14^ The Japan Coma Scale (JCS) was used to assess the level of consciousness,^15^ whereas the performance status (PS) was used to define the level of independence.^16^ The Functional Oral Intake Scale (FOIS) was used to assess the nutritional status.^17^

### Statistical analysis

The bedside evaluation and video recording evaluation of the same dentist were compared. In addition, the evaluation of the dentist who had performed the bedside assessment on the video was compared with the evaluations of the dentist or nurse who had not performed bedside assessment. The reliability of the ratings was examined by calculating the intraclass correlation coefficient (ICC) for the OHAT total score and the agreement rate and weighted kappa coefficients for the sub-item scores. In addition, Spearman’s rank correlation coefficients were calculated for basic information, such as age, BMI, FOIS, CCI, JCS, PS, Alb, CRP, and Hb, as well as the OHAT total score, evaluated by the dentist at the bedside. Statistical analyses were performed using SPSS Ver. 25(IBM, Japan), with a significance level of 5% for all statistical analyses.

## Results

### Participant’s characteristics

Fifty patients (26 males and 24 females; mean age, 68.9 ± 11.7 years) were included in this study. Table 1 presents the patient characteristics. Ten (20.0%), 15 (30.0%), and 25 (50.0%) patients were aged ≤60 years, 61–70 years, and ≥71 years, respectively, indicating a large proportion of elderly patients.

**Table 1:** Participant characteristics.

	Mean ± SD	Median	n	％
Age	68.9 ± 11.7	70.0	50	
Sex				
Male			26	52
Female			24	48
BMI (kg/m^2^)	21.0 ± 6.7	20.1		
FOIS	3.2 ± 2.1	3.0		
1			17	34
2			0	0
3			20	40
4			1	2
5			2	4
6			1	2
7			9	18
CCI score	3.1 ± 2.3	3.0		
JCS level				
0			28	56
Ⅰ			16	32
Ⅱ			4	8
Ⅲ			2	4
PS				
0			0	0
1			5	10
2			2	4
3			34	68
4			9	18
Alb	2.5 ± 0.6	2.6		
CRP	3.2 ± 4.0	1.4		
Hb	9.6 ± 1.3	9.7		

Abbreviations: Alb, albumin; BMI, body mass index; CCI, Charlson Comorbidity Index; CRP, C-reactive protein; FOIS, Functional Oral Intake Scale; Hb, hemoglobin; JCS: Japan Coma Scale; PS: Performance status; SD, standard deviation.

### Oral health assessment at the bedside and on the video recordings

Table 2 presents the results of the OHAT scores of the participants included in this study. The evaluation on the score of the lips on the video recordings tended to be high than at the bedside. On the video, the evaluation of the nurse on the score of saliva tended to be high, while that of dentists on the score of oral cleanliness tended to be high.

**Table 2:** OHAT individual sub-item scores and total score of the dentist who conducted oral health assessment at the bedside (B), the dentist who conducted oral health assessment at the bedside using the video recordings (VD1), the other dentist who conducted oral health assessment using the video recordings (VD2), and the nurse who conducted oral health assessment using the video recordings (VN).

Category	B	VD1	VD2	VN
	Mean ± SD	Mean ± SD	Mean ± SD	Mean ± SD
	Median	Median	Median	Median
Lips	0.5 ± 0.5	0.6 ± 0.5	0.5 ± 0.5	0.5 ± 0.5
	0.0	1.0	1.0	1.0
Tongue	0.7 ± 0.4	0.7 ± 0.4	0.8 ± 0.4	0.7 ± 0.6
	1.0	1.0	1.0	1.0
Gums and tissues	0.3 ± 0.5	0.2 ± 0.5	0.2 ± 0.5	0.1 ± 0.3
	0.0	0.0	0.0	0.0
Saliva	0.3 ± 0.4	0.2 ± 0.4	0.2 ± 0.4	0.5 ± 0.6
	0.0	0.0	0.0	0.5
Natural teeth	0.6 ± 0.7	0.5 ± 0.6	0.5 ± 0.7	0.4 ± 0.6
	0.0	0.0	0.0	0.0
Dentures	0.6 ± 0.9	0.5 ± 0.9	0.5 ± 0.9	0.5 ± 0.9
	0.0	0.0	0.0	0.0
Oral cleanliness	0.8 ± 0.7	0.8 ± 0.6	0.9 ± 0.6	0.6 ± 0.7
	1.0	1.0	1.0	0.5
Dental pain	0.0 ± 0.2	0.0 ± 0.2	0.0 ± 0.2	0.0 ± 0.2
	0.0	0.0	0.0	0.0
Total score	3.7 ± 2.1	3.6 ± 2.1	3.7 ± 2.1	3.6 ± 2.2
	3.5	4.0	3.0	4.0

Abbreviations: SD: standard deviation.

### Correlation between the OHAT total score at the bedside and basic information

Spearman’s rank correlation coefficients were calculated for the OHAT total score, as evaluated by the dentist at the bedside, and age, BMI, FOIS, CCI, JCS, PS, Alb, CRP, and Hb. The correlation coefficients for age, FOIS, JCS, and PS were r = 0.321 (*P* = 0.023), r = −0.498 (*P* < 0.001), r = 0.361 (*P* = 0.010), and r = 0.329 (*P* = 0.020), respectively, indicating a significant correlation.

### Reliability of oral health assessment performed using video recordings

The ICC of the OHAT total score was 0.914 (95% CI, 0.854–0.950) when the bedside assessment and video recording assessment of the same dentist were compared. Table 3 presents the percent agreement and weighted kappa coefficients for the sub-item scores. The agreement rate and weighted kappa coefficients for all sub-item scores were high, at >80% and >0.7, respectively.

**Table 3:** Percent agreement and weighted kappa coefficients for the individual OHAT sub-item scores and the intraclass correlation coefficient for the OHAT total score comparing the same dentist’s bedside (B) and video (VD1) evaluations.

Category	B - VD1	
	Percent agreement	Weighted Kappa Coefficients (95% CI)
Lips	88	0.761^a^(0.584–0.938)
Tongue	94	0.848^a^(0.682–1.014)
Gums and tissues	86	0.738^a^(0.501–0.976)
Saliva	90	0.722^a^(0.499–0.944)
Natural teeth	88	0.706^a^(0.456–0.957)
Dentures	96	0.896^a^(0.756–1.036)
Oral cleanliness	82	0.767^a^(0.620–0.914)
Dental pain	100	1^a^(1.000–1.000)
	Intraclass correlation coefficient (95% CI)	
Total score	0.914^a^(0.854–0.950)	

Abbreviations: CI: confidence interval.

a *P*＜0.05.

Comparison of the evaluation of the dentist who had performed bedside video assessment with the evaluations of the dentist and nurse who had not performed bedside video assessment revealed that the ICC of the OHAT total score was 0.904 (95% CI, 0.838–0.944) and 0.802 (95%CI, 0.676–0.883), respectively. Table 4 presents the agreement rates and weighted kappa coefficients for the sub-item scores. Relatively high agreement and weighted kappa coefficients of >70% and >0.5, respectively, were observed for all items for dentists. Agreement rates of >70% and weighted kappa coefficients of >0.5 were obtained for the dentist and the nurse for the sub-item scores of lips, tongue, natural teeth, dentures, and dental pain.

**Table 4:** Percent agreement and weighted kappa coefficients for the individual OHAT sub-item scores and the intraclass correlation coefficient for the OHAT total score of comparing the evaluation of the dentist who conducted oral health assessment at the bedside using the video recordings (VD1) with the evaluations of the other dentist (VD1) or the nurse (VN) who conducted oral health assessment using the video recordings.

Category	VD1 - VD2		VD1 - VN	
	Percent agreement	Weighted Kappa Coefficients (95% CI)	Percent agreement	Weighted Kappa Coefficients (95% CI)
Lips	88	0.759^a^(0.578–0.939)	86	0.717^a^(0.523–0.911)
Tongue	88	0.654^a^(0.404–0.903)	82	0.650^a^(0.464–0.836)
Gums and tissues	88	0.762^a^(0.527–0.997)	66	−0.171 (-0.269–0.073)
Saliva	90	0.700^a^(0.458–0.943)	64	0.406^a^(0.224–0.587)
Natural teeth	76	0.561^a^(0.293–0.830)	74	0.650^a^(0.456–0.845)
Dentures	94	0.880^a^(0.735–1.025)	94	0.880^a^(0.735–1.025)
Oral cleanliness	90	0.838^a^(0.697–0.979)	62	0.553^a^(0.355–0.751)
Dental pain	100	1^a^(1.000–1.000)	100	1^a^(1.000–1.000)
	Intraclass correlation coefficient (95% CI)		Intraclass correlation coefficient (95% CI)	
Total score	0.904^a^ (0.838–0.944)		0.802^a^(0.676–0.883)	

Abbreviations: CI: confidence interval.

a *P*＜0.05.

## Discussion

The results of the present study suggest that the OHAT total and sub-item scores showed high agreement when the same dentist conducted the oral health assessment at the bedside and on video in patients with malnourishment who were eligible for NST aid at an acute care hospital. Dentists and different professionals tended to show high agreement rates for the OHAT total score when oral health assessment was performed by different evaluators on video; however, the sub-item scores tended to be divided according to occupation. Thus, a comprehensive assessment of the oral health could be conducted using short video recordings acquired with a tablet device, regardless of the evaluator’s profession. This suggests that it may be possible to assess the dental issues of each patient using a video recorded via a tablet device even by NSTs with limited participation of dentists. This study is clinically significant in that it indicates the possibility of the clinical application of video recordings of the oral health acquired using a tablet device as a remote oral health assessment tool.

Poor oral health is associated with aging, nutritional intake, level of consciousness, and oral care techniques, which may reflect the degree of independence.^4^^,^^5^^,^^18^^,^^19^ The results of the present study indicated the presence of an association between oral health and age, nutritional intake, level of consciousness, and level of independence among patients requiring NST aid in acute care hospitals.

Nurses receive several opportunities to interact with patients in clinical practice. However, factors such as the lack of human resources and time constraints hinder oral health management by nurses.^20^^,^^21^ Therefore, to realize oral health assessment using videos in clinical settings, it is necessary to determine the usefulness of oral health assessment performed using videos acquired via means and methods suited to clinical settings. The results of the present study suggest the possibility of conducting oral health assessment using video recordings with the same reliability as that of bedside oral health assessment. The video recordings used for oral health assessment in this study were 1-min recordings of the oral health acquired with a tablet device, which is widely used in clinical settings and was shorter than the recordings used in a previous study.^12^ The results of this study indicate that the clinical application of oral health assessment using short videos acquired with tablet devices is possible.

Previous studies using the OHAT total score have reported significantly higher 3-month mortality rates in patients with empyema with an OHAT total score of ≥7,^22^ significantly higher 60-day mortality rates in patients admitted to acute care hospitals with an OHAT total score of ≥3,^23^ and significantly shorter survival rates in patients receiving palliative care with an OHAT total score of ≥6.^24^ Thus, the OHAT total score may be used as an indicator to determine the health status of patients. The agreement rates of the OHAT total score for oral health assessment performed using video recordings were high between dentists and between the dentist and the nurse. Thus, the OHAT total score can be used for comprehensive video oral health assessment by multiple professionals. An OHAT total score of 4 can be used as a cut-off value for referral to dentists while screening patients receiving NST aid in acute care hospitals;^25^ thus, the results of this study are clinically important as the use of a multidisciplinary OHAT total score assessed using video recordings may aid in identifying the requirement for dental intervention, even in cases where dental professionals are unable to directly assess the oral health status.

Low agreement was observed between the dentist and the nurse in terms of the gums and tissues, saliva, and oral cleanliness when the OHAT sub-item scores were determined via video evaluations. This tendency was consistent with the low agreement observed between dentists and nurses in their assessment of the oral health status in the study by Tsukada et al.,^26^ which may be attributed to the differences in the perception of oral health among different occupational groups. Dentists evaluate oral health based on the requirement for dental intervention; however, only 30% of nurses are trained in providing oral health care.^10^ Moreover, 50% of nurses cited a lack of guidelines and insufficient knowledge as barriers to providing oral health care,^21^ thereby suggesting that nurses are unable to accurately determine the differences between good and poor oral health status. Therefore, a short video, in which equal assessment among dentists was achievable, may be used to educate multiple professions on appropriate oral health assessment.

This study has certain limitations. First, almost all participants were able to communicate, which made video recording and listening easier. However, it is possible that some hospitalized patients or those who require home-visit treatment may be uncooperative. Second, a limited number of videographers and oral health evaluators were included in this study. Videos were captured by a dentist who was accustomed to oral examination, which may have made it easier to conduct the oral health assessment. Furthermore, the nurse who conducted the oral health assessment on the videos was a nurse certified in dysphagia nursing and had relatively more opportunities to evaluate the oral health status in routine practice. Thus, future, studies must expand the scope of the study participants and include videographers and oral health evaluators who are not accustomed to evaluating patients’ mouths. Third, video recordings in this study were acquired using limited means and techniques. Although the iPad Pro was used in this study, investigating the usefulness of oral health assessment performed using videos recorded with other devices, such as smartphones, is important.

In conclusion, the OHAT total score and its sub-items showed high agreement when a single dentist conducted oral health assessment at the bedside and using short video recordings of the oral health acquired with a tablet device in patients with low nutritional status who required the aid of NST. Furthermore, the OHAT total score showed high agreement when different evaluators conducted oral health assessment using the videos, regardless of occupation.

## Declaration of competing interest

The authors have no conflicts of interest relevant to this article. The funders had no role in designing the study; the collection, analyses, or interpretation of data; in the writing of the manuscript; or in the decision to publish the results.
